# High frequency ultrasound vibrational shear wave elastography for preclinical research

**DOI:** 10.1088/1361-6560/aca4b8

**Published:** 2022-12-07

**Authors:** J Civale, V Parasaram, JC Bamber, EJ Harris

**Affiliations:** 1 Division of Radiotherapy and Imaging, The Institute of Cancer Research, Sutton, United Kingdom

**Keywords:** ultrasound, preclinical imaging, shear wave elastography, xenograft tumour

## Abstract

Preclinical evaluation of novel therapies using models of cancer is an important tool in cancer research, where imaging can provide non-invasive tools to characterise the internal structure and function of tumours. The short propagation paths when imaging tumours and organs in small animals allow the use of high frequencies for both ultrasound and shear waves, providing the opportunity for high-resolution shear wave elastography and hence its use for studying the heterogeneity of tissue elasticity, where heterogeneity may be a predictor of tissue response. Here we demonstrate vibrational shear wave elastography (VSWE) using a mechanical actuator to produce high frequency (up to 1000 Hz) shear waves in preclinical tumours, an alternative to the majority of preclinical ultrasound SWE studies where an acoustic radiation force impulse is required to create a relatively low-frequency broad-band shear-wave pulse. We implement VSWE with a high frequency (17.8 MHz) probe running a focused line-by-line ultrasound imaging sequence which as expected was found to offer improved detection of 1000 Hz shear waves over an ultrafast planar wave imaging sequence in a homogenous tissue-mimicking phantom. We test the VSWE in an *ex vivo* tumour xenograft, demonstrating the ability to detect shear waves up to 10 mm from the contactor position at 1000 Hz. By reducing the kernel size used for shear wave speed estimation to 1 mm we are able to produce shear wave speed images with spatial resolution of this order. Finally, we present VSWE data from xenograft tumours *in vivo*, demonstrating the feasibility of the technique in mice under isoflurane sedation. Mean shear wave speeds in the tumours are in good agreements with those reported by previous authors. Characterising the frequency dependence of shear wave speed demonstrates the potential to quantify the viscoelastic properties of tumours *in vivo*.

## Introduction

Imaging is an attractive tool for monitoring tumour response to cancer therapy as it can map physical, functional or molecular properties of the entire tumour, as opposed to biopsy which only samples small volumes of tissue. Clinically, it can allow non-invasive assessment of tumour response, at an early stage, improving patient management (Brindle 2008). Preclinically, xenograft tumour models are a vital tool allowing researchers to test novel cancer therapies and imaging can be used to non-invasively assess the treatment at multiple time points, providing greater insight into the progression (or regression) of disease, whilst improving animal welfare and reducing animal numbers (Workman *et al* 2010). The identification of imaging biomarkers of tumour response that may be used both clinically or preclinically, requires *in vivo* validation (O’Connor *et al* 2008). Here preclinical tumour models, offer the opportunity for direct correlation of imaging signals with histological correlates of tumour biology.

It is now understood that elastic properties or the tumour play an important role in the development of cancer. For example, increased elastic modulus is characteristic of many solid tumours, and a driver of cancer progression (Kalli and Stylianopoulos 2018) and as such the elastic modulus of tissue is established as a diagnostic and prognostic biomarker of cancer (Runel *et al* 2021), and there is increasing evidence to suggest these properties may be useful as biomarkers of response to cancer therapy (Li *et al* 2014a).

Ultrasound shear wave elastography (SWE) uses measurements of the characteristics of shear waves propagating through tissue to determine shear wave speed. A characterisation of shear wave speed dispersion may then be used to characterise tissue viscoelastic properties, for example by quantifying the complex shear modulus. Both the frequency and amplitude of the shear waves used are important to consider. Shear waves must have sufficient amplitude to generate tissue displacements that are detectable using ultrasound. The minimum detectable displacement depends on the system imaging characteristics. Higher frequency shear waves (with shorter wavelengths) can improve the spatial resolution of SWE (Palmeri *et al* 2010, Rouze *et al* 2012, Tzschätzsch *et al* 2015), however, the attenuation of shear wave amplitude increases with frequency limiting the distance from the shear wave source over which oscillations can be detected.

Common methods used to generate shear waves in tissue include the use of an acoustic radiation force impulse (ARFI) or an external vibrational source. ARFI typically generates broad-band shear waves (Nightingale *et al* 2003) with properties which depend on the acoustic and geometrical properties of the impulse, but also on the properties of the tissue with which the ARFI beam interacts. The upper limit of ARFI shear wave spectrum in tissue is usually limited to less than 500 Hz (Tanter *et al* 2008, Mitri *et al* 2011). Preclinical applications of ARFI-based elastography include single tracking location shear wave elastography imaging (Ahmed *et al* 2020a) in pancreatic tumour liver metastasis, and harmonic motion imaging methods using the acoustic radiation force to generate localised vibrations at a frequency of 50 Hz in liver metastasis (Payen *et al* 2016). SWE in preclinical studies has been implemented with a high frequency probe (15 MHz) to characterise breast tumour stiffness in a xenograft model (Chamming’s *et al* 2013), and subsequently to study the response to anti-angiogenic therapy (Chamming’s *et al* 2016). Elastography techniques using external vibration sources include sonoelastography (Taylor *et al* 2000), where a reduction in vibration amplitude was correlated to increases tissue stiffness in excised prostate tissue (Taylor *et al* 2005), and SWE in mammary tissue (Wang and Insana 2013), with vibration frequencies restricted to less than 500 Hz.

The use of external vibration sources enables more control of the excitation, with narrow-band single or multiple frequencies, of relatively high amplitudes. In the context of preclinical imaging, in which tumours are small (typically <10 mm) and superficial, there is opportunity to use high frequency shear waves that have been generated by an external source. High spatial resolution SWE is desirable to be able to discern spatial heterogeneity in the elastic properties of tumours as it is well established that tumours are not homogenous, and heterogenous response to therapy drives treatment failure (Bedard *et al* 2013). Despite the small dimensions of preclinical tumours, attenuation of high frequency shear waves presents challenges.

The aim of the work described in this paper was to assess the feasibility of vibrational shear wave elastography (VSWE) for preclinical imaging systems. To image the finer detail of tumours a sufficiently high ultrasound imaging frequency is needed to provide the required spatial resolution. A high vibration frequency is also potentially advantageous in detecting spatial inhomogeneity in tissue mechanical properties. For these reasons we present a VSWE system using a high centre frequency ultrasound imaging probe, with vibration frequencies in excess of 500 Hz using shear waves generated by a single external vibration source. In this paper we begin by comparing VSWE implementations that do not require coherent compounding, namely an unsteered ultrafast planar wave against a custom designed scanned focused beam imaging sequence compatible with a high frequency continuous monochromatic shear wave signal. To assess the penetration depths at which high frequency shear waves can be detected, we quantify and map shear wave signal conformance, a metric that quantifies the percentage of the detected oscillation which is accounted for by the shear wave (and vibrational source) frequency. Subsequently we use the focused beam sequence to present shear wave data obtained in xenograft tumour models, both *ex vivo* and *in vivo*, demonstrating the feasibility of VSWE in estimating shear wave speed using high vibrational frequencies of between 500 and 1000 Hz.

## Materials and methods

### Test phantom and tumour model

VSWE imaging sequences were tested in a breast elastography biopsy phantom (model 059, CIRS, VA, USA). A homogeneous background region of the phantom was identified and imaged using simple planar wave, and focused beam imaging. According to the manufacturer’s specification the Young’s modulus in the background region of the phantom was 20 ± 5 kPa, equivalent to a shear wave speed of 2.6 ± 0.4 m s^−1^ assuming a mass density of 1000 kg m^−3^ and an isotropic incompressible perfectly elastic material.

The feasibility of VSWE was tested in xenograft tumour models. Xenograft tumours were grown following subcutaneous injection of cancer cells into the right flank of athymic nude mice (26 g weight). An initial *ex vivo* feasibility study was performed using an *ex vivo* tumour grown from C33A cell line. When the tumour reached a maximum diameter of 10 mm the mouse was terminated and the tumour was immediately imaged using VSWE at four frequencies (500, 600, 800 and 1000 Hz). *Ex vivo* imaging of the tumour allowed an initial assessment of the potential of the focused beam shear wave imaging sequence without the influence of breathing motion. Subsequently, three separate tumours grown in separate mice injected with MDA-MB-231 cells line were imaged using VSWE at three different frequencies (500, 700 and 1000 Hz) *in vivo* to assess performance of VSWE *in vivo*. Mice were anaesthetised with 2% isoflurane inhalation, and placed on a heated pad to maintain a temperature of 38°C during imaging. All animal experiments were approved by The Institute of Cancer Research Animal Welfare and Ethical Review Body, and performed in accordance with the UK Home Office Animals (Scientific Procedures) Act 1986, the United Kingdom National Cancer Research Institute guidelines for the welfare of animals in cancer research and reported according to the ARRIVE (animal research: reporting *in vivo* experiments) guidelines.

### Shear wave generation

**Figure 1.**
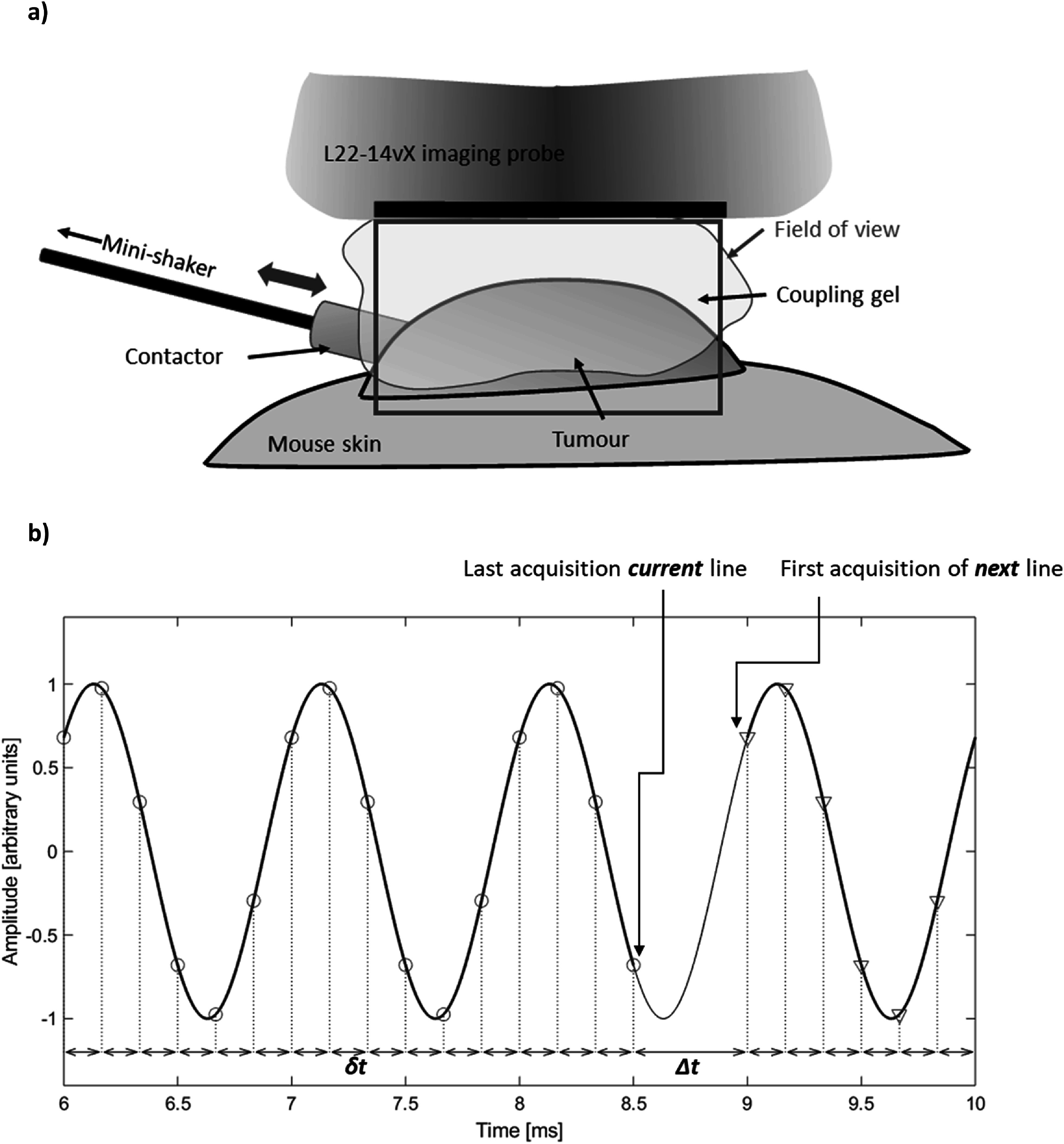
Diagram illustrating the experimental setup for vibrational shear wave elastography in the xenograft tumours (a); and schematic indicating the delays between transmissions events for the line-by-line imaging sequence for a 1000 Hz shear wave signal (b). The continuous black line indicates the harmonic shear wave signal at fixed location. A regular short delay, δt, is used to sample a section (blue line) of the shear wave oscillations at regular intervals (6× shear wave frequency). A longer time delay, Δt, is used between the last transmission of the current A-line, and the first transmission event of the next A-line, so phase coherence is maintained between A-lines. The value of Δt is automatically calculated based on the number of samples to be acquired, the value of δt, and the shear wave frequency.

Shear waves were generated in phantom and the tumour by coupling vibrations from a mini shaker (model 4810, Bruel & Kjaer, Denmark) via a 4 mm thick cylindrical carbon fibre rod placed in physical contact with the skin overlying the tumour or to the outer surface of the phantom or tissue being imaged (figure 1(a)). The shaker was supported by a manual precision stage (Thorlabs, UK) allowing precise positioning (<20 *μ*m) of the contactor. A minimal amount of pressure was applied at the contactor to maintain physical contact at the surface of the phantom or tumour skin. The carbon fibre rod and contactor were oriented horizontally parallel to and aligned with the ultrasound imaging plane. Due to the relatively large size of the phantom the contactor remained a small distance away (15–20 mm) from the lateral edge of the ultrasound field of view. The nature of the subcutaneous tumours is such that a large enough portion of it protrudes above the skin of the surrounding tissues, allowing positioning of the contactor on the lateral side of the tumour in the gap between the imaging probe and the skin of the tissue surrounding the tumour. Due to the small size of the tumour, it is possible to visualise the contactor in the ultrasound imagine field of view. The shaker was driven by a narrowband continuous signal (up to 1000 Hz) generated by a signal generator (Agilent 33120 A, UK) connected to a 500 W audio amplifier (Intimidation VLV-1000, UK) with independent gain control.

### US imaging system and shear wave detection

Ultrasound imaging was performed using a Vantage 256 imaging system (Verasonics Inc., Kirkland, WA, USA) in conjunction with a high frequency (17.8 MHz nominal centre frequency) L22-14vX imaging probe (Verasonics Inc.). The performance of the imaging system was initially tested in a CIRS breast elastography phantom, (model 059, CIRS Inc., Norfolk, VA, USA). The Vantage system’s image reconstruction software was used to compute image In-phase/Quadrature (IQ) data allowing visualisation of B-mode images and computation of shear waves oscillations. Ultrafast imaging with unsteered planar transmitted waves was compared to a conventional scanned focused beam sequence modified for tracking of shear wave oscillations at high frequency. The rationale for this comparison was to determine whether ultrafast imaging with planar wave unfocused transmission was capable of adequately detecting shear wave oscillations, when compared to a focused beam approach. Synthetic focusing methods, for example coherent compounding using multiple steered planar transmissions, were not considered at this time. For the planar wave sequence the imaging depth was set to 12 mm with a pulse repetition frequency set to an exact multiple (10×) of the shear wave vibration frequency for a total of 200 frames of IQ data. The modified focused beam line-by-line sequence consisted of 52 repeat transmission events for each A-line to sample shear wave oscillations, before moving to the next A-line in the sequence. A total of 128 separate sets of A line transmissions and line reconstructions were performed for an imaging depth of 12 mm. The transmit focus was set to 7 mm depth, with a transmit aperture size of 1 mm. The focused beam sequence shares some similarity to stroboscopic techniques used to track shear waves (Nightingale *et al* 2003, Gennisson *et al* 2004). The pulse-echo repetition frequency was set to 6 times the vibration frequency. A longer delay in transmission events was implemented between the last transmission event of an A-line, and the first transmission event of the next A-line to maintain shear wave phase coherence between acquisition of successive A-lines (figure 1(b)). The duration of the longer delay necessary between A-lines was calculated and set automatically according to the sampling parameters and vibration frequency to maintain phase coherence with the shear wave vibrations at source. A full set of 128 A-line data sets were collected this way, and transferred to the host computer for IQ data reconstruction. Phantom measurements using the different imaging sequences were performed immediately after another maintaining the same contactor position and excitation voltage. A comparison of data acquisition parameters between planar wave imaging and focused line-by-line imaging is given in table 1. In order to maximise the number of cycles that could be acquired in the line-by-line imaging sequence, the number of samples per cycle (6×) and the vantage’s receive bandwidth were both reduced (50% versus 200%) when compared to the planar wave imaging sequence. These steps were necessary to maximise the temporal duration and hence number of cycles that could be acquired for the line-by-line imaging sequence.

**Table 1.** Parameters for the planar wave and line-by-line imaging sequence used in the study for an imaging depth of 12 mm and a shear wave frequency of 1000 Hz. Receive bandwidth indicates the verasonics vantage system’s sampling frequency setting, where 200% is equivalent to 4 samples per wavelength, and 50% to 2 samples for every 2 wavelengths.

	Planar wave imaging	Line-by-line imaging
Vibration frequency (Hz)	1000	1000
Number of vibration cycles/A-line	20	8
Pulse repetition frequency (kHz)	10	6
Number of temporal samples/A-line	200	52
Number of A-lines	128	128
Total Number of transmissions events	200	6656
RF sampling bandwidth	**200%**	**50%**
RF samples/A-line/transmission	**1408**	**352**
Total RF samples/A-line	**281 600**	**23 42 912**
Total acquisition time (ms)	**20**	**1152**

The focused beam line-by-line and planar wave imaging sequences were evaluated in terms of signal to noise ratio. Rectangular and Hanning window transmit apodisation functions were tested for planar wave imaging. To compute signal to noise ratio images, the VSWE sequences were run to obtain a set of IQ data frames without any vibration for each transmission method. One set of data was obtained whilst imaging a homogeneous section of the CIRS breast elastography phantom, and another whilst imaging degassed water in the absence of any scatterers to compute the signal and noise estimates respectively. Signal to noise ratio was quantified in dB using

\begin{eqnarray*}{\mathrm{SNR}}=10{\mathrm{log}}_{10}\left({P}_{S}/{P}_{N}\right),\end{eqnarray*}

where *P*_*S*_ and *P*_*N*_ represent the power in the IQ data for the signal and noise measurements. Power in the IQ data sets was computed by multiplying the IQ data with its complex conjugate at each pixel, followed by temporal and local spatial (0.8 mm square) averaging.

The reconstructed image IQ data was processed in a similar way as reported by Ormachea *et al* (Juvenal *et al* 2018). A 2D phase axial velocity estimator (Loupas *et al* 1995) was applied to the IQ data to compute local tissue axial velocities, amplitude and phase data at the shear wave frequency were obtained from a Fast Fourier transform applied in the temporal domain to the local tissue shear velocity data. The conformance, *C*, of the detected shear wave was calculated as the percentage of the total detected vibrational energy that was measured at the shear wave drive frequency:

\begin{eqnarray*}C\left( \% \right)=\displaystyle \frac{100\,{\left|{S}_{f}\right|}^{2}}{\displaystyle \sum {\left|{S}_{i}\right|}^{2}},\end{eqnarray*}

where ∣*S*_*i*_∣ represents spectral amplitude components, and ∣*S*_*f*_∣ is the amplitude component at the shear wave frequency. The detection of shear waves depends on the amplitude of the oscillation being large enough to be detected, but also on the signal to noise ratio of the ultrasound echo data. The conformance measure therefore represents a simple and effective method to visualise detection of shear waves that combines the factors above. A further sanity check is provided by inspecting the shear wave phase images, allowing the user to confirm the shear wave field that is being generated conforms to the expected form, for example shear waves propagating away from the contactor position.

Shear wave speed was estimated from the shear wave phase images using a square shaped kernel based 2D autocorrelation approach similar to that used by Parker *et al* (2017). Prior to shear wave speed estimation, spatial median filtering was applied to the detected shear wave field data using a 2 × 3 pixel kernel to reduce the effect of impulse noise. Our method differs from the method used by Parker *et al* (2017) in that it assumes non-reverberant, locally-planar shear wave propagation, and the kernel size can be smaller than the shear wavelength, allowing for localised estimation of shear wave speed. The 2D autocorrelation data is used to determine the local direction of the shear wave in the region defined by the kernel. Subsequently, a 1D cosine curve is fitted only to the real part of the autocorrelation data aligned with the direction of the shear wave. The relatively small size of the kernel therefore inherently allows for any variation in shear wave propagation through the imaging plane due to diffraction. The cosine fitting process yields a shear wave wavelength estimate, *λ*. The wavelength, and vibration frequency *f* are then used to calculate the shear wave speed, *c*:

\begin{eqnarray*}c=\lambda \,f.\end{eqnarray*}

## Results

**Figure 2.**
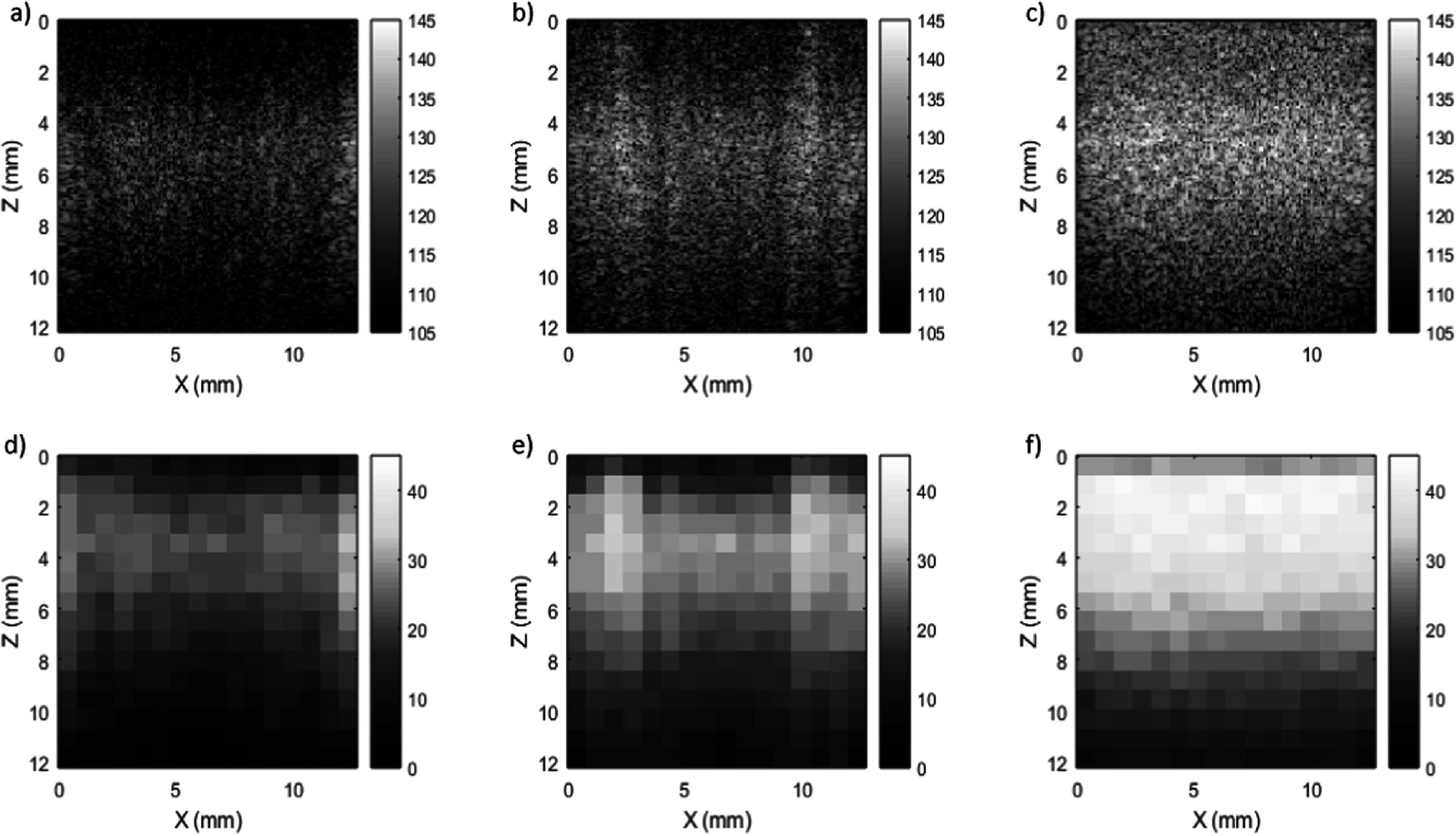
B-mode images (a)–(c) and signal to noise ratio images [dB] (d)–(f) of a uniform section of the CIRS breast elastography phantom imaged with the L22-14vX corresponding to planar wave imaging with no transmit apodisation (a), (d), planar wave imaging with Hanning transmit apodisation (b), (e) and focused beam line-by-line imaging (c), (f).

B-mode images from a homogeneous section of the CIRS elastography phantom are shown in figures 2(a)–(c) for planar wave imaging with rectangular and Hanning transmit apodisations, and with the focused line-by-line sequence. Amplifier gain was maintained constant, and the contactor position was left unchanged between the different imaging scans. The images show inhomogeneous brightness across the image for both planar wave imaging sequences. The focused beam method however shows a higher and more uniform level of brightness across the image. Figures 2(d)–(f) show the signal to noise ratio measurements for the imaging sequences, quantifying the variations that were observed in the B-mode images. The highest signal to noise ratios were obtained using the focused beam line-by-line imaging sequence which showed a consistent improvement, typically between 10 and 15 dB, in signal to noise ratio with depth over the planar wave transmission with rectangular apodisation sequence. Applying Hanning windowing to the planar wave transmission improved signal to noise ratio by approximately 5 dB. The echogenicity and signal to noise ratio using the focused beam imaging was also found to be more homogeneous across the lateral direction when compared to the planar transmissions.

**Figure 3.**
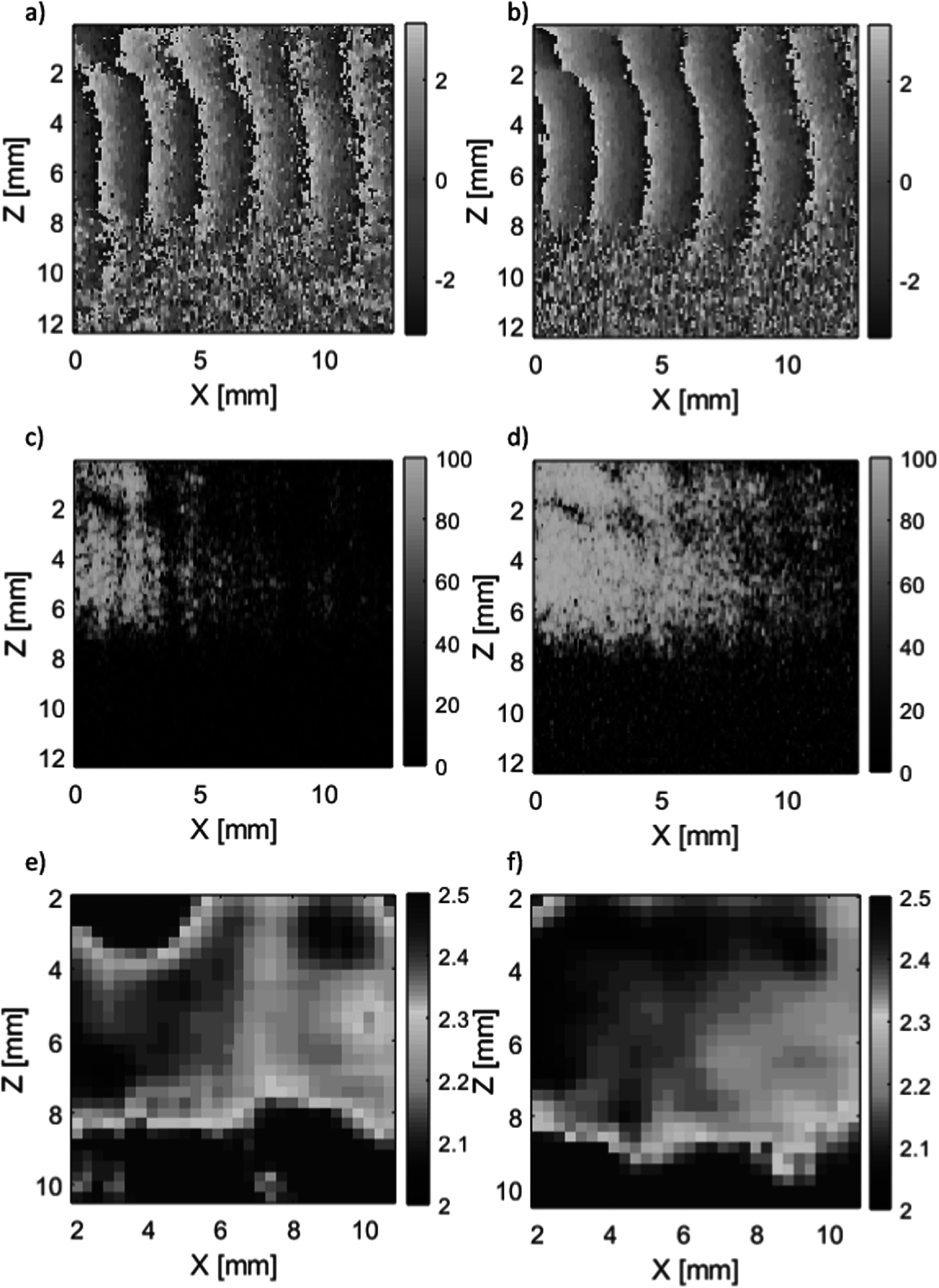
Detected 1000 Hz shear wave oscillation phase [rad] (a), (b), conformance [%] (c), (d), and estimated shear wave speed [m s^−1^] (e), (f) for planar wave imaging with Hanning transmit apodisation (a), (c), (e) and focused beam imaging (b), (d), (f) in the CIRS phantom.

The CIRS breast phantom was also used to evaluate the detection of 1000 Hz shear waves generated in the phantom. For these measurements the contactor was positioned aligned with, but just outside, of the imaging plane so that shear waves could be detected travelling across the imaging plane. Figure 3 shows a comparison of the phase, conformance and shear wave speed images obtained using planar wave imaging with Hanning transmit apodization, and the focused line-by-line sequence. The phase images (figures 3(a), (b)) show the progressive wave nature of the shear waves. The improved ultrasound signal to noise ratio using the focused line-by-line sequence is reflected in the relative appearance of the shear wave phase images, and the greater extent of higher conformance values (>80%) at positions further away from the contactor. These images illustrate the improved detection of the shear wave oscillations using the focused beam imaging method as an improvement in the apparent penetration depth of the shear wave. The shear wave speed maps show a mostly homogeneous distribution across the phantom with the exception of the most distal region from the probe, and for the planar wave imaging, a region close to the transducer surface with elevated shear wave speed compared to the surroundings (figures 3(e), (f)). The increased shear wave speed in the distal region can be understood as arising due to the poor signal to noise ratio in this region due to attenuation of the ultrasound echoes. The mean and standard deviation of the shear wave speed images were 2.24 ± 0.21 and 2.32 ± 0.25 m s^−1^ for the focused beam and planar wave imaging respectively. If only the middle third section of the phantom only is considered, eliminating the proximal and distal regions to the probe, the mean and standard deviation were found to be 2.15 ± 0.06 and 2.17 ± 0.07 m s^−1^ for the focused beam and planar wave imaging respectively. These results are close to the shear wave speed expected (2.3–2.9 m s^−1^) given the manufacturer’s specification of a Young’s modulus of 20 ± 5 kPa for the phantom material, assuming an elastic isotropic material with a mass density of 1000 kg m^−3^. The results are also in good agreement with the values reported by Ormachea *et al* (Juvenal *et al* 2018) on a similar phantom.

**Figure 4.**
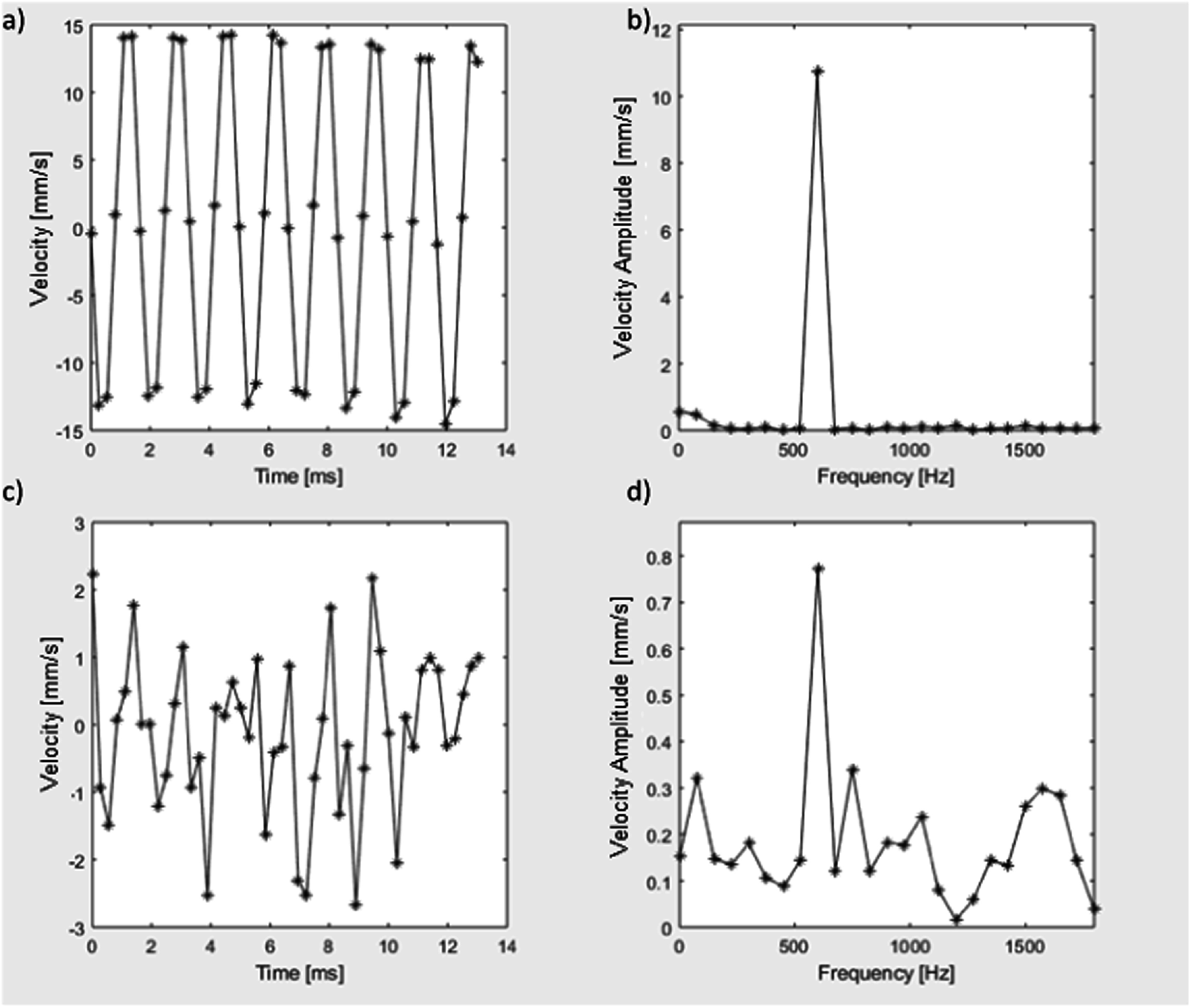
Detected 600 Hz shear wave oscillations: time domain (a) and spectrum (b) for location with signal conformance greater than 99%; time domain (c) and spectrum (d) for location with signal conformance of 60%.

The improved performance of the focused beam line-by-line imaging sequence over simple planar wave imaging was not surprising since the latter is also known to be more susceptible to grating lobe artefacts (Montaldo *et al* 2009). Vibrational SWE was therefore tested in the xenograft *ex vivo* tumour using the focused beam imaging sequence. An example of the detected shear wave oscillations is given in figure 4, comparing the time domain signal and spectra from regions with high (>99%) and low (60%) conformance.

**Figure 5.**
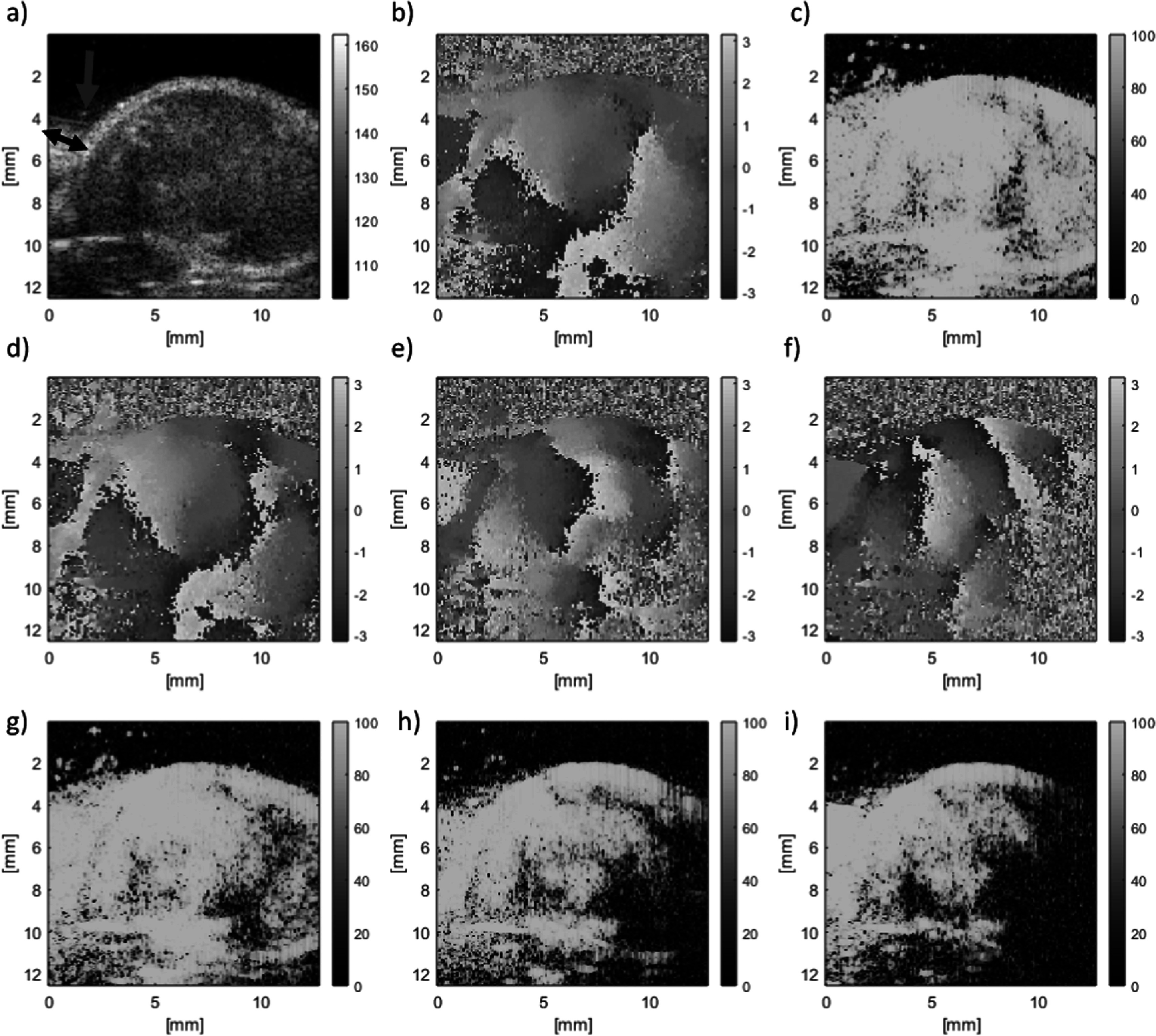
Shear wave detection in an *ex vivo* tumour with focused beam imaging. Example B-mode image with the contactor indicated by the red arrow (a) and the direction of motion of the contactor (black arrow), phase [rad] (b), and conformance [%] (c) with a shear wave frequency of 500 Hz. Phase images (d)–(f), and conformance images (g)–(i) are shown for 600, 800 and 1000 Hz shear wave frequencies respectively.

Examples of phase and conformance images obtained in the *ex vivo* tumour are shown in figure 5, for vibration frequencies from 500 Hz to 1000 Hz. With the contactor placed on the tumour skin, as indicated in figure 5(a), it is possible to visualise the penetration of the shear wave into the tumour. Higher amplifier gain settings were required at the higher frequencies. Amplifier gain was increased within a safe operating limit to improve detection of the shear waves, as determined by inspection of the real-time conformance image. At a frequency of 500 Hz shear wave oscillations are detected throughout the tumour. As the frequency is increased the conformance images show a progressive reduction in the ability to detect shear wave oscillations due to increased shear wave attenuation. At 600 Hz conformance is reduced towards the distal edge of the tumour with respect to the position of the contactor. At 800 Hz the shear waves do not propagate further than 8–10 mm from the contactor, and at 1000 Hz it is difficult to detect any shear waves beyond 10 mm from the contactor. The phase images in the tumour show a progressive shear wave similar to the ones observed in the CIRS phantom at the higher frequencies of 800 and 1000 Hz. At the lower frequencies there is more evidence of possible interactions between the shear wave propagating into the tumour reflections from the tumour surface.

**Figure 6.**
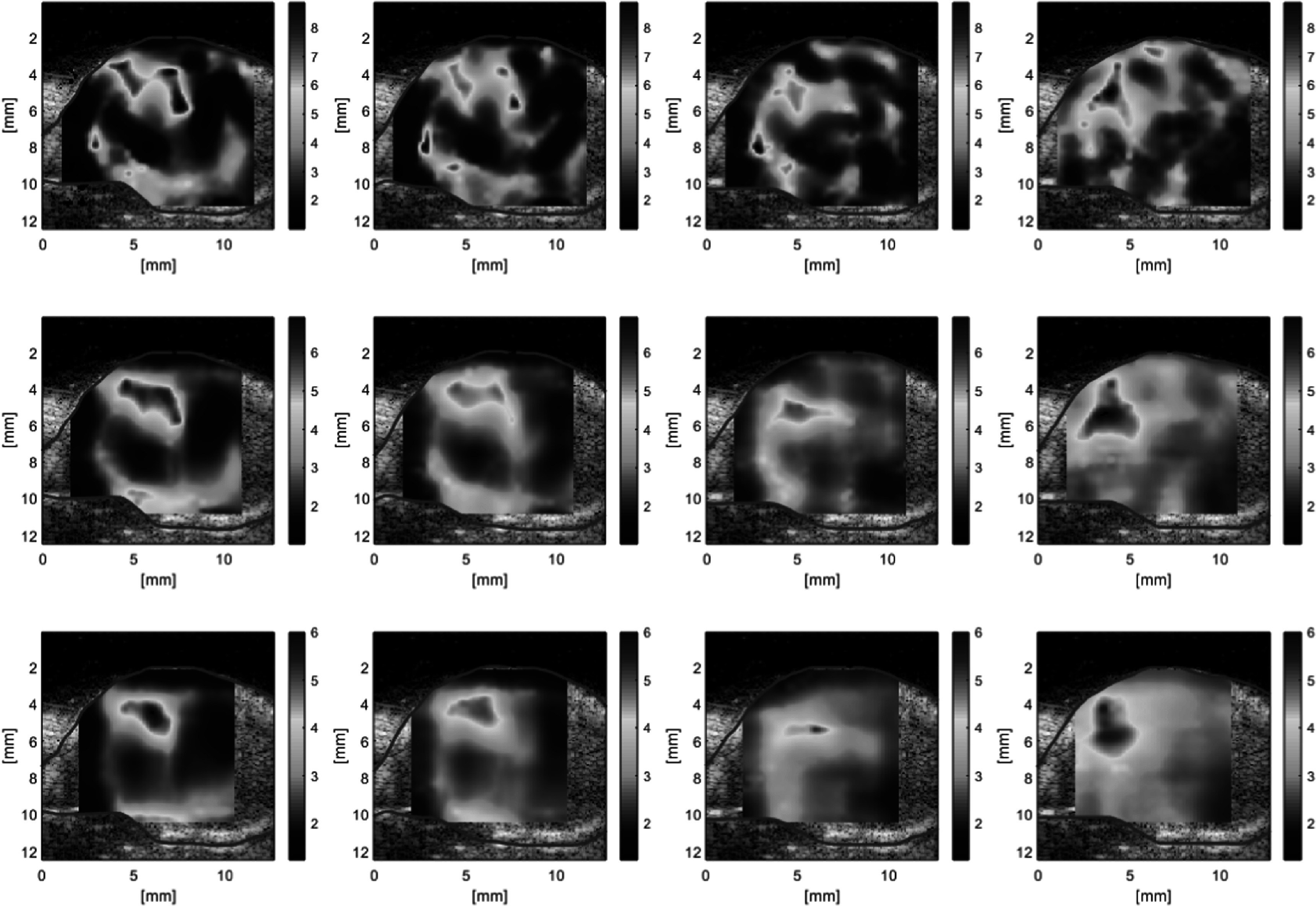
Estimated shear wave speed [m s^−1^] in *ex vivo* tumour for frequencies of 500, 600, 800 and 1000 Hz (columns, left to right), using a 1 × 1 mm, 2 × 2 mm and 3 × 3 mm kernel (rows, from top to bottom).

**Figure 7.**
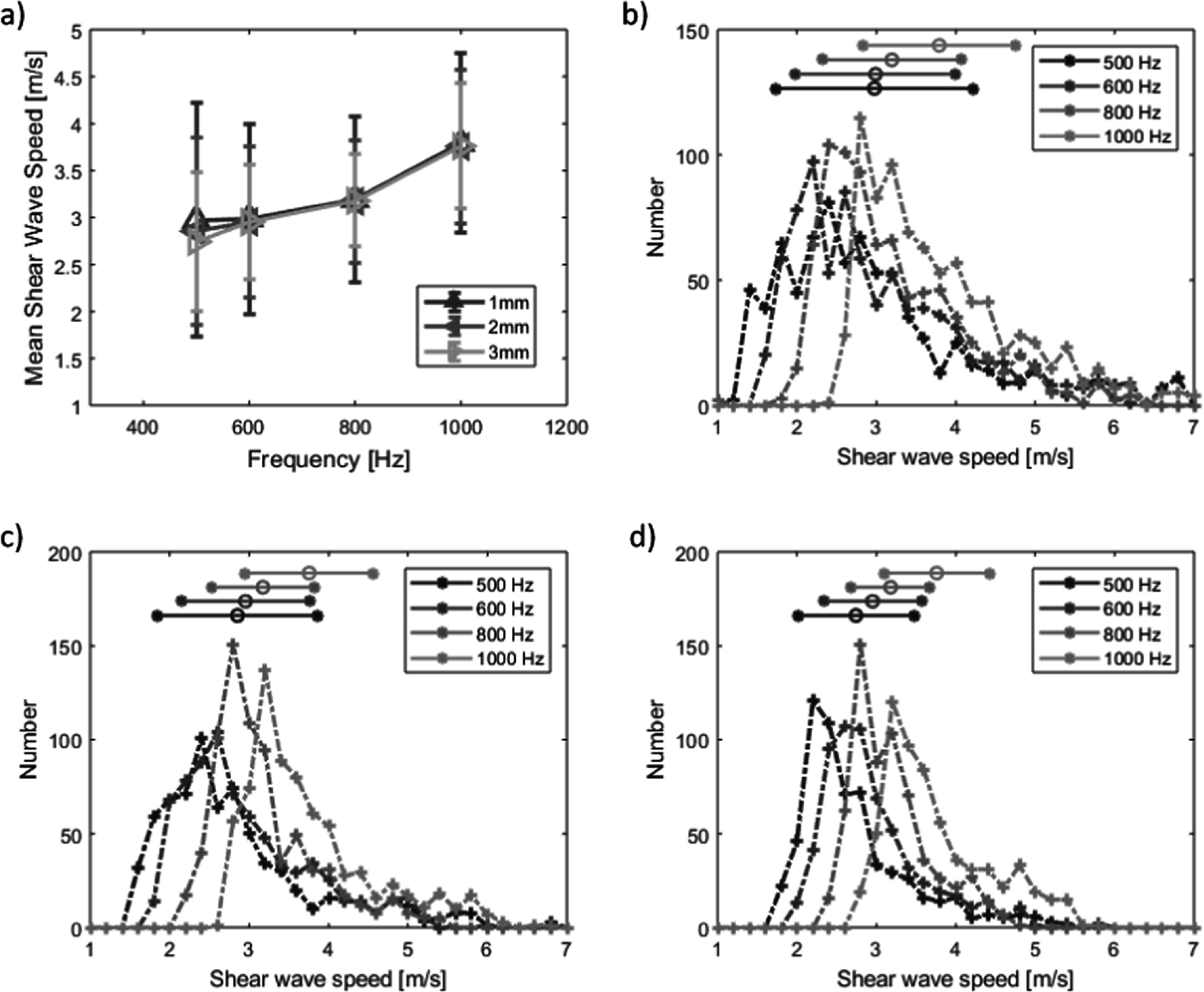
Mean shear wave speed in *ex vivo* tumour versus vibration frequency estimated with 1 × 1 mm, 2 × 2 mm and 3 × 3 mm kernel, error bars indicate standard deviation (a). Shear wave speed histograms using 1mm (b), 2mm (c) and 3mm (d) square kernels for shear wave speed estimation, horizontal bars represent the mean ± one standard deviation for each vibration frequency.

The appearance of the tumour in B-mode imaging was used to segment the shear wave speed images to show data in the tumour region only. The shear wave speed images computed using the three different kernel sizes show the appearance of an apparent stiffer region in the upper part of the tumour (figure 6). The mean shear wave speed in the tumour increases with frequency for all three kernel sizes (figure 7(a)). The main effect of varying kernel size is to alter the spatial resolution of the final shear wave speed image. The smaller kernel size (1 mm) produces more detailed images but is more susceptible to noise. Moving to a larger kernel size increases the area used for spatial averaging in computing the autocorrelation and is an effective way to reduce some of the variance in the shear wave speed image. A comparison of the mean and standard deviation of the shear wave speeds at different frequencies for the different kernel sizes (figures 7(b)–(d)) illustrates the reduction of the variance in shear wave speed as the kernel size is increased.

**Figure 8.**
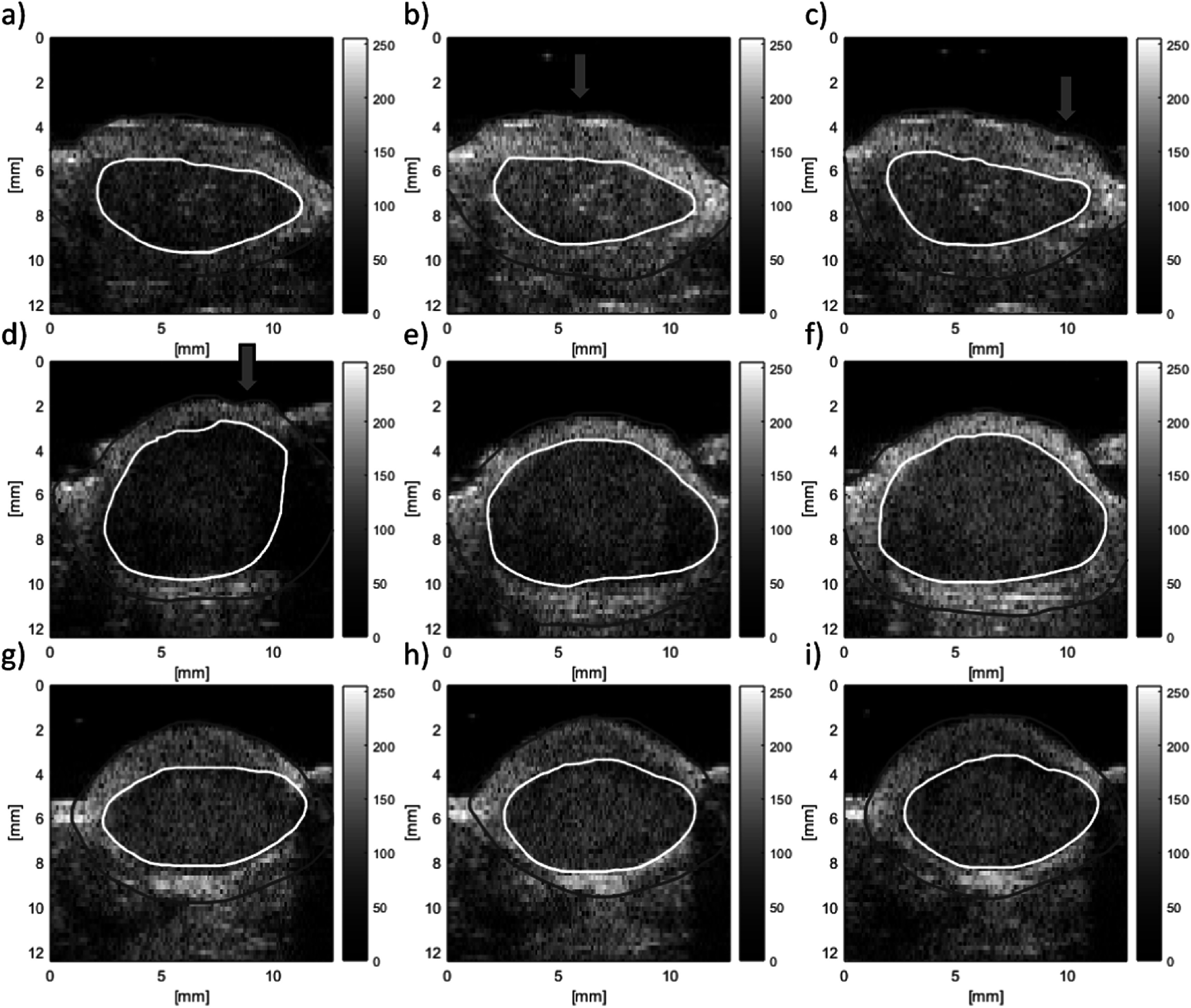
B-mode (a.u.) outline of three tumours (rows) imaged *in vivo* using VSWE. Red outline includes the whole tumour including hyper-echoic rim, white outline indicates body of the tumour. Columns represent images obtained at 500, 700 and 1000 Hz vibration frequency respectively. The contactor was positioned towards the top right of the tumour in all images. The effect of tissue motion can be observed by the apparent undulation of the skin surface, examples of which are indicated by the green arrows.

**Figure 9.**
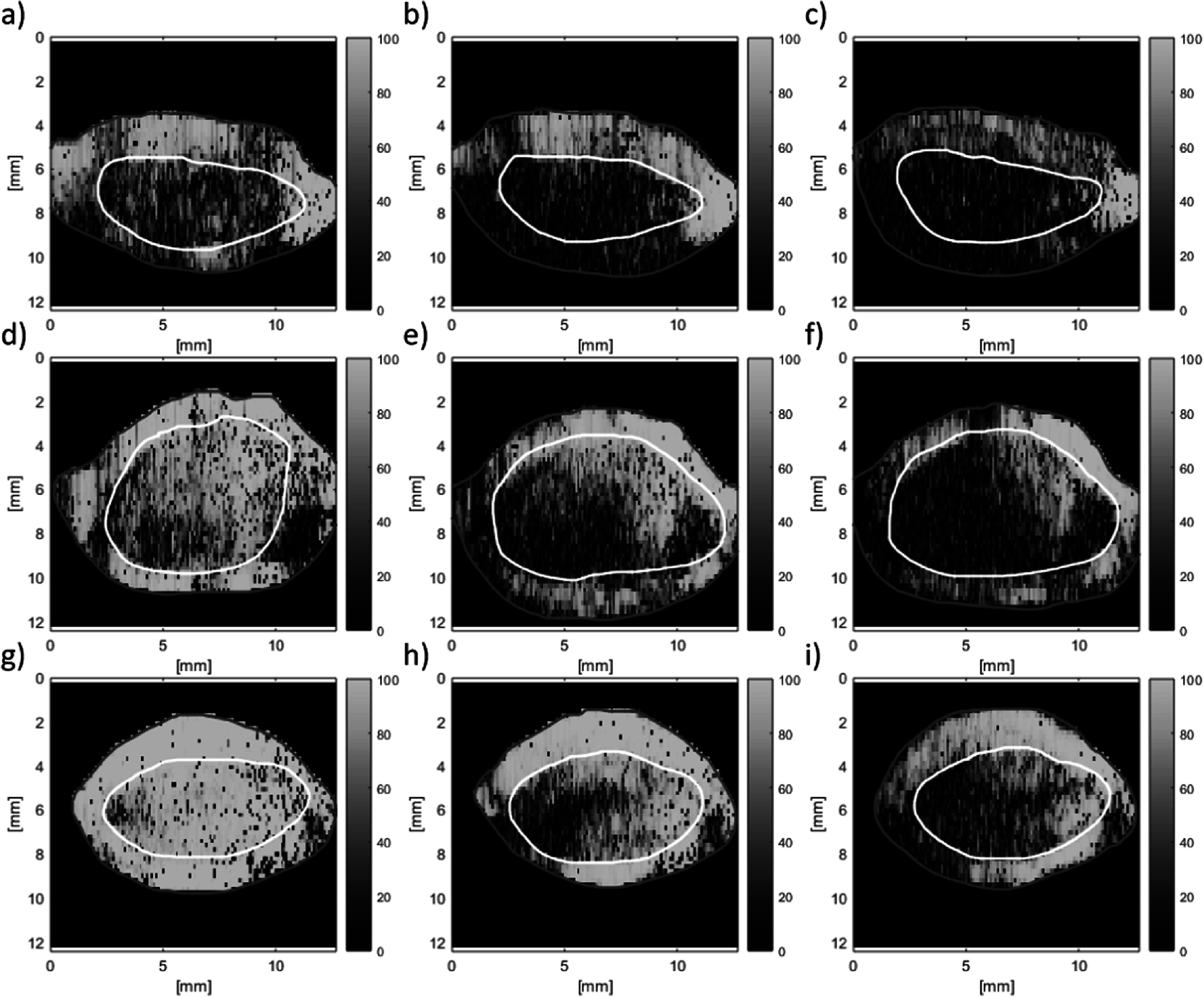
VSWE conformance (%) images for *in vivo* tumours corresponding B-mode images shown in figure 8. conformance images represent different tumours (rows) and vibration frequencies (columns) of 500, 700 and 1000 Hz respectively. Outlines are those drawn using the B-mode images, as indicated in figure 8.

**Figure 10.**
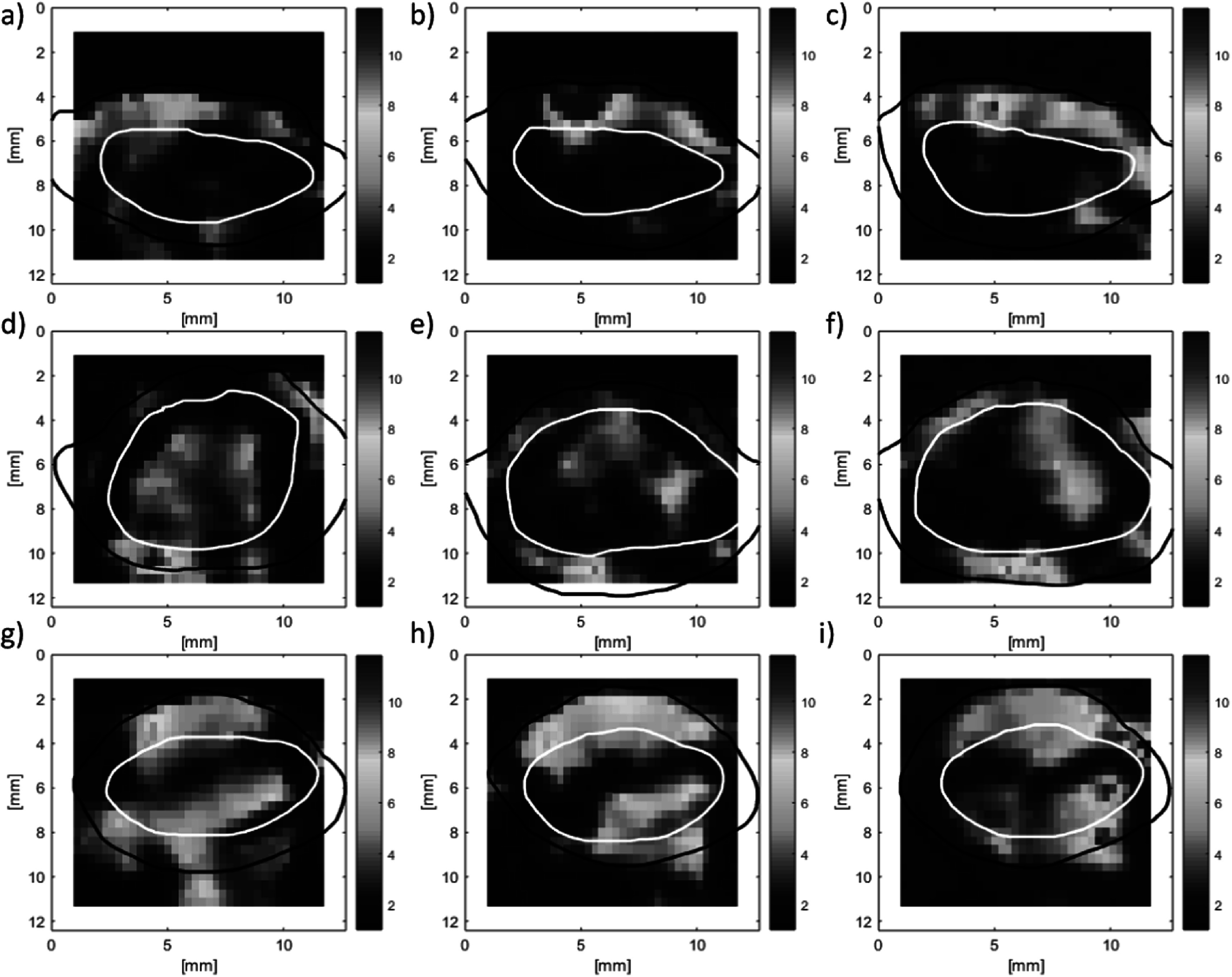
VSWE shear wave speed (m s^−1^) images for *in vivo* tumours corresponding to the B-mode and conformance images shown in figures 8 and 9 respectively. Shear wave speed imaged represent different tumours (rows) and vibration frequencies (columns) of 500, 700 and 1000 Hz respectively. Outlines are those drawn using the B-mode images, as indicated in figure 8.

Finally, VSWE was tested *in vivo* with the focused beam sequence on xenograft tumours in mice under sedation with isoflurane. The B-mode images of three tumours at three vibration frequencies are shown (figure 8), with their respective conformance (figure 9). Outlines of the tumour including a hyper-echoic rim around the edge (red) and the body of the tumour (white) are included. The *in vivo* conformance images replicate the *ex vivo* findings, namely that at 500 Hz it is possible to generate and detect shear vibrations throughout the tumour, and increasing the frequency reduces the effective penetration of the shear waves within the body of the tumour. At the higher frequency of 1000 Hz shear waves were mostly detected along the top (figures 9(c), (f) and (i)) and bottom of the tumour (figure 9(i)). A visualisation of the detected shear wave phase temporal evolution for the three tumours at each frequency can be observed in the supplementary video. These data were used to compute shear wave speed using a 1 mm kernel size (figure 10) which reveals regions of greater shear wave speed consistent with the location of the upper rim, including the skin, of the tumour for mouse 1 and mouse 3 (figures 10(a)–(c), (g)–(i)). Analysis of the shear wave speed data obtained within the tumour body (white outline in figure 10) reveals mean and standard deviations in shear wave speed of 1.9 ± 0.8, 2.5 ± 1.2 and 3.4 ± 1.6 m s^−1^ in the three tumours respectively (table 2). The relatively large variation of shear wave speed within each tumour (up to 50%) is driven by localised regions of increased shear wave speed that are broadly consistent across the three measurement frequencies.

**Table 2.** Summary of mean shear wave speed (±standard deviation) measured in the body and rim of three *in vivo* tumours.

SWS in tumour body (m s^−1^)				
	500 Hz	700 Hz	1000 Hz	Mean
Mouse 1	1.9 ± 0.8	1.8 ± 0.9	2 ± 0.8	1.9 ± 0.8
Mouse 2	2.6 ± 1.1	2.4 ± 1.1	2.6 ± 1.3	2.5 ± 1.2
Mouse 3	3.4 ± 1.4	3.4 ± 1.7	3.5 ± 1.7	3.4 ± 1.6

SWS in tumour rim (m s^−1^)				
	500 Hz	700 Hz	1000 Hz	Mean

Mouse 1	2.9 ± 1.5	3.1 ± 2.7	3.3 ± 1.9	3.1 ± 2.0
Mouse 2	2.5 ± 1.9	2.6 ± 1.3	3.3 ± 1.6	2.8 ± 1.6
Mouse 3	3.4 ± 1.7	4.1 ± 2.1	4.1 ± 2.3	3.9 ± 2.0

## Discussion

To characterise shear wave speed in tumours in preclinical studies using SWE, high frequencies are desirable for both the ultrasound imaging and for the shear wave vibration source in order to detect and visualise small (<∼2 mm) features in the tumours. In practice this requires ultrasound imaging frequencies at high frequencies (15 MHz and above), and vibration frequencies approaching 1000 Hz to obtain shear wavelengths of only a few (<∼5 mm) millimetres. We have demonstrated the feasibility of a vibrational SWE method implemented with a focused beam, line-by-line, scanning method. Our approach using the conventional focused beam line-by-line method to reconstruct the image and detect the shear waves has some similarity to the work published previously by others (Tzschätzsch *et al* 2018, Rabin and Benech 2019, Huang *et al* 2020). Our method differs from those of previous authors in that we take advantage of the continuous nature of the high frequency harmonic vibration which generates a steady state (non-transient) shear wave field. Unlike sequential A-line acquisition used in standard B-mode imaging, here we repeatedly scan individual A lines enabling the use of high pulse repetition frequency to avoid aliasing the high frequency shear waves. Subsequent A-lines transmissions are then synchronised in such a way that phase coherence is maintained at the shear wave frequency, eliminating the need for phase-unwrapping.

The focused beam method was found to produce image data with improved signal to noise ratio (>10 dB) and more homogeneous echogenicity when compared to unsteered planar transmit waves. Line-by-line focused beam imaging was chosen here as an alternative to multi-angle, steered, spatial compounding methods because it represented a simple and efficient way to achieve transmit focusing and maximise image quality. It has been shown by Montaldo *et al* (2009) that images with similar quality to those obtained with focused beam method can be obtained using synthetic focusing by coherent spatial compounding with multiple steered planar transmissions. Indeed, an advantage of coherently adding images obtained with planar wave transmissions over multiple angles is that the overall frame rate compares favourably with the focused beam approach providing an increase of approximately an order of magnitude in overall frame rate. A higher frame rate is a desirable feature in a preclinical ultrasound elastography system where the temporal duration of anaesthesia is an important consideration, however it should not significantly compromise the ability to accurately map shear wave oscillations. A limiting factor of coherent spatial compounding methods is the degree of motion, due to shear wave oscillations or otherwise, that can result in loss of coherence (Denarie *et al* 2013, Ahmed *et al* 2020b). This is likely to be an important consideration when imaging high frequency oscillations (1000 Hz) where in order to adequately track the oscillations a choice has to be made in the trade-off between imaging depth, number of compounded images, and effective frame rate. A study comparing the relative merits of the focused beam imaging method versus synthetic focusing with coherent compounding methods, likely to require motion correction (Denarie *et al* 2013), is beyond the scope of this paper and will be the subject of future studies.

The focused beam approach places greater demands on the Vantage 256 system’s memory for image data reconstruction (table 1). Therefore, to maximise the number of temporal samples of the shear wave oscillations that could be acquired for a single frame, data reduction in the digitised RF signal was achieved by lowering the Vantage receive bandwidth setting (50% versus 200%). The reduced bandwidth did not produce any observable image degradation effects in mapping shear wave oscillations. The number of temporal samples (52) acquired per image line using this method was sufficient to sample up to 8 cycles of the shear wave oscillations with the minimum possible of 6 samples per cycle compatible with the IQ phase change detector (Loupas *et al* 1995) used to track the shear oscillations. This choice proved adequate for monochromatic shear waves as described here, and is potentially sufficient to acquire shear waves signals containing several distinct harmonic frequencies.

The increase in ultrasound echo signal to noise ratio using the focused beam line-by-line, compared to planar wave imaging, is thought to be one of the principle factors in the improvement in shear wave phase images (figure 3). This improvement was quantified in terms of higher conformance of the detected shear wave signals, leading to an increase of approximately 5 mm in 1000 Hz shear wave penetration depth in the CIRS phantom.

The focused beam line-by-line imaging sequence was therefore used to characterise the shear wave fields generated in an *ex vivo* xenograft tumour model. Measurements were performed over a range of vibration frequencies to investigate the nature of the shear waves fields that may be generated using a single contactor. At low frequencies (500 Hz) it was possible to detect shear wave oscillations throughout the tumour with good conformance levels (>80%). As the vibration frequency was increased, the penetration of the shear wave was reduced due to attenuation, and the nature of the shear wave began to more clearly resemble that of an attenuated progressive wave. At 1000 Hz vibration frequency the penetration of the shear wave was limited to within 10 mm from the contactor position, rendering characterisation of the shear wave speed difficult in the regions distal to the contactor. Shear wave speed images computed from acquisitions at different frequencies indicate an overall increase in the shear wave speed with frequency, providing a potential basis to quantify the viscoelastic properties of tissue. An important advantage of using ultrasound to characterise frequency dependent shear wave speed is the relative speed of the measurement compared to other modalities such as for example magnetic resonance elastography (Muthupillai *et al* 1996, Schregel *et al* 2020). In principle data could be obtained at several frequencies over the course of a few seconds using ultrasound imaging, whereas the long scan times (Schregel *et al* 2020, Runel *et al* 2021) associated with magnetic resonance imaging (> 20 min) make this unfeasible for *in vivo* applications.

In this study the choice of kernel size had an impact on the level of detail that can be observed in shear wave speed images. A small kernel size allows potential visualisations of small regions (1 mm) of the tumour, conversely increasing the kernel size has the effect of smoothing out some of the variance in the shear wave image if a mean value is required. In the example illustrated here, a small region with increased shear wave speed is visible towards the top of the tumour was observed at all frequencies and kernel sizes (figure 6). In the absence of an independent gold-standard measurement of tissue stiffness this feature roughly coincided with a hyper-echoic region in the B-mode image. For *in vivo* studies the choice of kernel size to be used is largely going to be dependent on a compromise between improved spatial resolution, with a small kernel size, versus the need to reduce variance by averaging over a wider area with a larger kernel in the presence of noisy shear wave data.

Our results using the focused beam approach represent an optimisation of imaging quality, using transmit focusing, at the expense of overall scanning speed, which was not a significant factor for phantom work and *ex vivo* tissue studies presented here. *In vivo* tissue motion may lead to artefacts in the accurate tracking of shear wave displacements, resulting in wave speed image artefacts or errors. For example, Ahmed *et al* (Ahmed *et al* 2020b) used ARFI based STL-SWE to measure shear wave speed in tumours in mice under isoflurane sedation. Their imaging method consisted of coherent compounding of the echoes from three angled planar transmit waves, and they found artefacts in the shear wave speed images which they associated with breathing motion inhalation events. To overcome this problem these authors monitored breathing motion and applied a gating technique to estimate shear wave speed only during ‘quiet’ times of the breathing cycle. To some extent the effect of breathing motion in our *in vivo* data can be observed in terms of the modulation of the skin surface apparent in the B-mode images (figures 8(b), (c), (d)). With the tumour positioned on the flank of the mouse and imaged from above, the breathing motion was predominantly aligned in a vertical directional parallel to the imaging beam axis, and approximately orthogonal to the direction of shear waves. Despite the presence of this breathing motion, which is therefore also parallel to the direction of detection of shear oscillations, it was still possible to detect local shear wave motion. The maximum tissue velocity associated with shear vibration at high frequency is comparable to maximum tissue velocity due to breathing motion, even for shear vibrations with displacement amplitude of only a few micrometres. It is possible to distinguished the shear wave oscillation from breathing motion by considering their respective spectra. Over the short time duration (∼10 ms) where tissue velocity is tracked, the breathing motion with relatively low frequency content (≪100 Hz) manifests as a near constant value or drift velocity, whereas the shear oscillations appear as a clear peak in the spectrum at the drive frequency (≥500 Hz). This spectral filtering approach also ensures that it is possible to extract shear wave amplitude and phase in the presence of noisy signals where conformance is low. Under these conditions and with mice under isoflurane sedation, VSWE results using the focused beam sequence were not significantly compromised by tissue motion without the need to apply any breathing motion gating. Whilst effort was made to image the central part of the tumour, measurements at different frequencies were taken a few minutes apart (<5 min), hence small differences in tumour appearance or outline between these measurements are thought to be due to small movements of the animal under isoflurane sedation between scans.

The nature of the shear wave fields generated in xenograft tumours is potentially complex. At 500 Hz where shear wave attenuation is expected to be lower there is possibility of interaction between the outgoing shear wave from the contactor and reflections arising from within the body and boundaries of the tumour. The presence of surface waves (Rayleigh waves) near the boundaries of the tumour is also possible. The wave speed associated with surface waves is not quantitatively equal to that of shear waves travelling in the bulk, where surface waves may be approximately 5% lower than the shear wave speed in the bulk of the tumour for a viscoelastic soft tissue (Royston *et al* 2011). The penetration of surface waves within the tumour would be expected to be less than one wavelength from the boundary, and therefore will be dependent on both the exact wave speed and vibration frequency. For data presented here the penetration of surface waves could potentially be as large as 5 mm for the combination of shear wave speed and frequency of 3 m s^−1^ and 500 Hz respectively. It was not possible to clearly distinguish between surface Rayleigh waves and true shear waves travelling through the body of the tumour. Another factor to consider is the nature of the contact between the tumour and surrounding tissues, for example the skin. The conformance images also suggest the shear vibrations, particularly at the higher frequency of 1000 Hz, travel around the outside of the tumour and through the skin more readily than within the body. This is not surprising as the skin is directly in contact with the external vibration source. These locations were also consistent with increased shear wave speed with respect to the body of the tumour immediately below (figure 10 and table 2). Vibration in the skin may be better described as guided waves (Lamb waves), which potentially include partial standing waves or components which travel perpendicular to the imaging plane leading to wave speed overestimates. Shear wave speed estimates in the body of the tumour showed relatively large standard deviations which were driven by localised regions of higher shear wave speed that were broadly consistent across vibration frequencies in each tumour. Regions of low shear wave speed (<2 m s^−1^) also contribute towards the variance, and visually correlate with regions of low conformance.

Despite the above limitations, and considering the range of factors potentially influencing the mechanical properties of xenograft tumours (collagen content, tumour location, extent of tissue necrosis), the shear waves speeds measured in the body of the tumours are in good agreement with values reported by other authors. Li *et al* (2019) used MR elastography to measure the complex shear modulus of xenograft tumours in mice at a vibration frequency of 1000 Hz. These authors also studied MDA-MB-231 tumours, and reported mechanical properties with equivalent shear wave speeds of 3.3 ± 0.2 m s^−1^ (orthotopic), and 2.2 ± 0.2 m s^−1^ (intracranial). The same authors reported complex shear modulus for other orthotopic tumours including BT-474 (2.9 ± 0.2 m s^−1^), PANC1 (3.3 ± 0.2 m s^−1^), and other intracranial tumours from GTML/Trp53 (2.1 ± 0.2 m s^−1^) to U87 MG (2.5 ± 0.2 m s^−1^), and finally subcutaneous SW620 (2.3 ± 0.2 m s^−1^) (Li *et al* 2014b). Page *et al* (2019), reported the shear elastic modulus of SA-LIV tumours using MR elastography at 600 Hz (1.3 m s^−1^). Ahmed *et al* (2020b) used STL-SWE, an ARFI based ultrasound shear wave imaging modality to measure the shear wave speed of PDAC xenograft tumours (2.3 ± 0.5 m s^−1^) in mice.

There is scope for further optimisation of VSWE by improving the coupling of vibration from the contactor to the tumour tissue, particularly for frequencies of 1000 Hz where conformance measures in the body of the tumour was low. This may potentially be achieved by optimising the contactor shape design. A measurement of shear vibrations in 3D, using a 2D array probe for example, and an improved understanding of the nature of shear vibrations in the tumour and surrounding tissue, by modelling or other means, are the next steps to improve characterisation of mechanical properties of preclinical tumours. *In vivo* results using small (≤3 mm) kernel sizes therefore suggest VSWE has potential for assessing heterogeneity in response to treatment by means of quantifying frequency dependent shear wave speed, and hence tissue viscoelastic properties.

## Conclusion

We have demonstrated the feasibility of high spatial resolution (<5 mm) measurements of shear wave speed using vibrational SWE in an human tumour xenograft model at high vibration frequencies (500–1000 Hz) *in vivo*. Vibrational SWE has therefore potential in quantifying frequency dependent shear wave speed, and hence tissue viscoelastic properties, providing potential analysis of tumour heterogeneity in response to treatment.

## Ethical statement

All animal experiments were approved by The Institute of Cancer Research Animal Welfare and Ethical Review Body, and performed in accordance with the UK Home Office Animals (Scientific Procedures) Act 1986, the United Kingdom National Cancer Research Institute guidelines for the welfare of animals in cancer research and reported according to the ARRIVE (animal research: reporting *in vivo* experiments) guidelines.
